# Perturbation of Brain Oscillations after Ischemic Stroke: A Potential Biomarker for Post-Stroke Function and Therapy

**DOI:** 10.3390/ijms161025605

**Published:** 2015-10-26

**Authors:** Gratianne Rabiller, Ji-Wei He, Yasuo Nishijima, Aaron Wong, Jialing Liu

**Affiliations:** 1 Department of Neurological Surgery, University of California at San Francisco and Department of Veterans Affairs Medical Center, 1700 Owens Street, San Francisco, CA 94158, USA; E-Mails: gratianne.rabiller@gmail.com (G.R.); jiwei.he@ucsf.edu (J.-W.H.); nishijima_yasu@yahoo.co.jp (Y.N.); aaronwong95@gmail.com (A.W.); 2 UCSF and SFVAMC, San Francisco, CA 94158, USA; 3 Univ. de Bordeaux, Institut des Maladies Neurodégénératives, UMR 5293, Bordeaux 33000, France; 4 CNRS, Institut des Maladies Neurodégénératives, UMR 5293, Bordeaux 33000, France; 5 Department of Neurosurgery, Tohoku University Graduate School of Medicine 1-1 Seiryo-machi, Aoba-ku, Sendai 980-8574, Japan; 6 Rice University, 6100 Main St, Houston, TX 77005, USA

**Keywords:** electroencephalography, action potential, MCAO, CBF

## Abstract

Brain waves resonate from the generators of electrical current and propagate across brain regions with oscillation frequencies ranging from 0.05 to 500 Hz. The commonly observed oscillatory waves recorded by an electroencephalogram (EEG) in normal adult humans can be grouped into five main categories according to the frequency and amplitude, namely δ (1–4 Hz, 20–200 μV), θ (4–8 Hz, 10 μV), α (8–12 Hz, 20–200 μV), β (12–30 Hz, 5–10 μV), and γ (30–80 Hz, low amplitude). Emerging evidence from experimental and human studies suggests that groups of function and behavior seem to be specifically associated with the presence of each oscillation band, although the complex relationship between oscillation frequency and function, as well as the interaction between brain oscillations, are far from clear. Changes of brain oscillation patterns have long been implicated in the diseases of the central nervous system including ischemic stroke, in which the reduction of cerebral blood flow as well as the progression of tissue damage have direct spatiotemporal effects on the power of several oscillatory bands and their interactions. This review summarizes the current knowledge in behavior and function associated with each brain oscillation, and also in the specific changes in brain electrical activities that correspond to the molecular events and functional alterations observed after experimental and human stroke. We provide the basis of the generations of brain oscillations and potential cellular and molecular mechanisms underlying stroke-induced perturbation. We will also discuss the implications of using brain oscillation patterns as biomarkers for the prediction of stroke outcome and therapeutic efficacy.

## 1. Introduction

Electroencephalography (EEG) has commonly been used as a non-invasive method of recording and analyzing electrical activity of the brain via electrodes attached to the scalp. This test is most often used to diagnose and monitor various neurological diseases including ischemic stroke and seizures. In particular, EEG has been instrumental in differentiating acute ischemic stroke from stroke mimics. This review summarizes the current knowledge of brain oscillatory wave changes recorded by either conventional EEG or penetrating electrodes during human or experimental stroke from extracellular recordings to molecular events. It will first describe the fundamentals and utility of using EEG in a normal mammalian adult brain, as well as discuss neural oscillations as being the primary basis of analysis of EEG. Next, it will focus on both how stroke conditions modify the brain oscillations typically observed in EEG and which biomarkers can be used to detect and predict these outcomes. While acknowledging the variability reported by different sources of literature regarding EEG changes after stroke, this review will conclude by considering both the molecular events that occur during ischemia and the structures that generate neural oscillations in an attempt to draw conclusions about brain oscillations and give a new approach to brain connectivity. Although most experimental data were collected by using penetrating electrodes instead of scalp EEG, the term EEG is still used in the relevant context throughout this review in order to make reference to the frequency groups originally identified by conventional EEG.

## 2. EEG Signals and the Spectrum of Oscillations

EEG is a widespread technique to study brain activity under physiological as well as pathological conditions. In humans, EEG records the electrical activity of the superficial layers of the brain using electrodes placed on the skull. Classically, the location of the electrodes is determined according to the “10–20 System of Electrode Placement” method that refers to a 10% or 20% inter-electrode distance of the total front-back or right-left distance of the skull. Electrodes are distributed on the scalp and identified by the first letter of the brain regions (e.g., F, T, C, P and O for frontal, temporal, central, parietal and occipital lobe) and electrode number (1, 3, 5, 7 assigned for the left hemisphere and 2, 4, 6, 8 for the right hemisphere). The letter Z usually refers to an electrode placed on the midline. The summation of the currents from cortical neurons can be detected by using two electrodes about 5 mm in radius that permit measurement of small current potential up to 100 µV [1]. Due to the simplicity of this approach, EEG is one of the most widespread non-invasive techniques for neural activity recording as a diagnostic tool for clinical purposes [2]. However, this technique does have some caveats that are mainly related to the tissue barrier of the scalp that prevents the detection of low-energy brain activity, such as frequencies higher than 100 Hz and those lower than 0.1 Hz. Furthermore, artifacts can be created by eye blinks, movements, or muscle activity such as respiration.

The utility of EEG as a diagnostic tool or in getting high-quality data is reduced when it comes to laboratory animals like rodents due to the following limitations: (1) lack of adequate space to accommodate the electrodes because of the small size of the rodent brains; (2) difficulty in locating the anatomic source of neural activity in epidural EEG recordings; and (3) lack of real time capability to extract signal characteristics due to the requirement of extensive computational analysis. To circumvent the first two limitations, the use of an invasive technique, such as probe insertion, permits exploration of the activity of deeper structures in the brain including the thalamus or hippocampus. In particular, the use of microelectrode arrays can register the activity of small groups of neurons, referred to as “local field potentials”, or a single neuron, known as “single-unit action potential”, with a signal frequency up to 5000 Hz. The electrode diameter inserted in the brain ranges from 10 to 30 µm, affording a great deal of tissue coverage up to 50 mm^2^ on average [3] and a high spatial resolution that is required to analyze the neural substrates for complex tasks. Despite this enhanced sensitivity and specificity, the downside of using these penetrating electrodes still remains due to the invasive aspect of this technique, as insertion of a probe several millimeters deep into the brain can destroy neurons along the pass [4].

By using penetrating and scalp electrodes, EEG has provided us with invaluable information regarding the generation, propagation, patterns and functions of brain oscillations for more than a century, with the first animal publication dating back to 1890 (by Adolf Beck [5]) and the first human investigation in 1929 (by Hans Berger [6]), respectively. It is our current understanding that brain oscillations resulting from electrical currents propagate in all mammalian brains within the frequency range of 0.05 to 500 Hz. For all intents and purposes, the oscillations are categorized into five main frequency groups, namely δ (1–4 Hz), θ (4–8 Hz), α (8–12 Hz), β (12–30 Hz) and γ (30–80 Hz) [7]. Apart from those commonly observed in the conventional EEG, there are other oscillations outside this spectrum. For example, there exist slow oscillations (0.3–1 Hz) that are slower than the δ band [8] and high frequency oscillations (HFO) (80–200 Hz) that are faster than the γ band, also known as fast oscillations that include ripples (100–200 Hz) [9]. Data from human sleep studies suggest that the slow (<1 Hz) and δ bands are two different oscillatory types that are distinct in their evolution; *i.e*., the power of the δ waves declined from the first to the second non-Rapid Eye Movement (REM) sleep episode, while the power of the slow wave remained unchanged [10]. Furthermore, pathological high frequency oscillations (pHFOs) (200–600 Hz) that are distinct from normal ripples are often recorded in the dentate gyrus during seizure generation [11]. It should be mentioned that the frequency of the θ band from superficial layers of the brain (4–8 Hz) differs from that recorded in the hippocampal layers (4–10 Hz) [12]. In addition, another oscillation band known as the mu rhythm (8–13 Hz) shares a great deal of similarity in frequency with that of the α band. However, unlike α which is recorded in the visual cortex in the occipital lobe, mu is not only recorded at various locations in the motor cortex such as the central and parietal areas, but also as a sinusoidal, regular, and rhythmic waveform that is distinct from the sharp negative peak and rounded positive phase observed in the α band. In the low frequency range, some confusion may arise due to inconsistent nomenclature in reference to the slow oscillations that exist during slow-wave sleep, anesthesia or after stroke and the δ oscillations present during slow-wave sleep or after stroke. Indeed, these two low frequency waves differ by their frequency range because the slow oscillations refer to activity between 0.3 and 1 Hz in an adult awake EEG [8] whereas the δ wave refers to activity between 1 and 4 Hz [13,14].

In order to determine the changes in brain oscillations associated with behavior-specific neural activity or pathological processes, it is critical to first understand the EEG patterns in a variety of normal physiological conditions including sleep, awake, immobile, and highly mobile states from various brain regions in the cortex, brainstem, thalamus, and limbic areas. The normal range of the EEG frequency, also called background activity, is around or above 8.5 Hz in the posterior head regions in awake adults. In contrast, the background activity is dominated by the β rhythm in the anterior brain regions, and by the β, α, and θ rhythms in the central and temporal regions, respectively. Due to rapid changes in EEG features during early development with respect to temporal and spatial organization and age-specific unique patterns in pediatric brains that are not linked to pathology, we will limit our discussion of this review to adult EEG only [15].

EEG translates a three-dimensional electrical wave into a two-dimensional electrical wave using two electrodes as reference points. Thus, an epoch of EEG recording represents a time-varying dynamic of voltage difference (*i.e*., potential in mV or µV) between two locations (e.g., a target site *vs.* reference/ground). EEG signals in the time domain often contain slow and fast oscillations, amplitudes of which wax and wane in a complex fashion; hence, the raw EEG information is not intuitive to the naked eye. As such, a Fourier transformation is frequently used to parcel out specific frequency bands simultaneously and to reveal the unique characteristics of the EEG from its complex time domain. As a frequency domain representation of the original data, the Fourier transformation provides information in the amplitude (mV or V) or power (mV^2^ or V^2^) of any frequency band over a period of time. In principle, data of a longer period generates a parcellation of frequency bands with finer resolution, and in turn results in a more precise estimate of amplitude at a given frequency. However, in practice, data of interest often do not last for a long time. Therefore, the parameters of the Fourier transformation are often dictated by specific scientific questions or the exact protocol that may vary between studies. The distribution of each wave throughout the entire brain under normal physiological conditions following the Fourier transformation spectrum excluding the γ band is as following: 25%–45% of δ oscillations, 40% of θ oscillations, 12%–15% of α oscillations, and 3%–20% of β oscillations in rodent EEG in the global frequency band (0–30 Hz) [16,17].

## 3. EEG in Normal Conditions

### 3.1. Generators of Oscillations

The EEG signal can be obtained by the volume conduction of the brain with the electrical current propagating from the generators to the recording electrode through brain tissue. Due to the physics of waves, slower oscillations propagate more than higher frequency ones, recruiting a larger network as in the case of θ and δ waves [18,19]. Although it is established that EEG records the currents from the cortical neurons, the exact origin of the electrical activity or intermediate partners involved in driving these events are not well understood. Because EEG translates a three-dimensional signal in a two-dimensional signal, it is not possible to precisely localize the electrical sources of the oscillations [20]. It is hypothesized that certain brain structures or neuronal networks serve as the generators of various oscillation frequencies similar to pacemakers, while others act like the resonators that respond to certain firing frequencies [21]. It appears that the locations of the generators may vary depending on the frequencies. For the slow-wave state present during non-REM sleep (frequency inferior at 1 Hz), the two main oscillation generators are located in the neocortex (pyramidal neurons in the layers II/III, V, and VI) and the thalamocortical (TC) and nucleus reticularis thalami (NRT) neurons in the thalamus. A synchronization is established between these two generators via corticothalamic, thalamocortical, and intracortical connections [22].

The generators of the θ wave have been proposed in several locations. To investigate deeper structures that can act as potential generators, electrode implants were particularly pertinent. One report suggests that structures like the entorhinal cortex and medial septum may act like pacemakers, inhibiting or exciting certain subregions of the hippocampus to synchronize the θ wave [12,23,24]. In comparison, the hippocampus acting like a resonator generates the θ oscillation that propagates via the volume conduction through the septo-temporal axis [25]. Hence, the inactivation or lesion of the septum perturbs the hippocampal θ oscillations [23]. However, a discrepant report implicated the source of the θ to originate from within the hippocampus (*i.e*., in the cornu ammonis 1 (CA1) and dentate gyrus (DG), propagating the current into the superficial and deep layers of the brain, respectively). Despite the fact that θ oscillation has also been observed in the perirhinal cortex, cingulate cortex, subiculum, and amygdale [26,27,28,29,30], these structures are generally not considered as proper generators but rather as resonators of the currents (dipoles) because they cannot generate θ activity by themselves.

The δ wave is generated by the thalamus and pyramidal cells located in layers II–VI of the cortex, whereas higher frequency oscillations like α or β are believed to be generated by the cells in layers IV and V of the cortex [31,32,33]. However, contradicting results raise the possibility that the α wave is generated from locations other than the cortex. For example, it is present in subcortical regions like the hippocampus or the reticular formation [34]. It is also prominent in the thalamus and can be seen in isolated thalamic networks [35]. Further evidence suggests that cortical α is driven by thalamic pacemaker cells [34] and the thalamo-cortical-thalamic network [36,37]. As a direct support for the thalamic origin of α, thalamic lesions lead to α rhythm disorganization or suppression in humans [38,39]. In addition, an occipital α rhythm episode is associated with an increase in the thalamic activity as measured by blood oxygenation [40,41] or blood flow [42].

The γ rhythm seems to be present in several different brain structures associated with visual, auditory, and motor tasks [43,44,45,46]. The cortical γ seems to be generated by the superficial layers II/III [33,47,48] and networks of interconnected inhibitory interneurons [49]. At the network level, tetanic stimulation of the thalamic reticular nucleus induces focal cortical γ oscillations via primary sensory pathways [50]. Further, following the stimulation of the pacemaker cells located in the reticular nucleus of the thalamus (another reported location of generator), there is an increase of the γ oscillation (35–55 Hz) in the somatosensory and auditory cortex [50]. An alternative school of thought suggests that γ oscillations are generated by synaptic activity via the interaction between neurons [51,52]. For example, γ oscillations can be generated by pacemaker cells located in the hippocampus that entrain the “chattering cells” in the cortex to fire at the same frequency [48]. *In vitro* studies have shown that the γ rhythm can be elicited in cortical and hippocampus slice preparations after stimulation of the metabotropic receptors for a long period of time [47] or by activation of metabotropic glutamate receptors with bursts of afferent stimulation for transient amounts of time [49,53,54]. Likewise, the subiculum can generate γ oscillations via the local inhibitory neuronal network following stimulation evoked either locally or in the nearby hippocampus CA1 [55].

### 3.2. Oscillations and Behavior

Since the EEG technique was invented, efforts have been made to understand the association between a specific brain oscillation and corresponding behavior with some success. This chapter provides an overview in the amplitude or power of dominant waves observed during a specific behavior in humans and in animals with either scalp EEG or inserted electrodes in deeper structures. We also highlight a different aspect of the cortical state known as the synchronized *vs.* desynchronized state, in addition to the classical view of oscillation defined by the frequency range.

#### 3.2.1. In Humans

Slow oscillations (0.3–1 Hz) and δ oscillations (1–4 Hz) are present during anesthesia and slow-wave sleep, suggesting their roles in the consolidation of neuronal connections and new memories acquired during wakefulness [56]. Increased amplitude in the δ wave has also been detected after auditory target stimuli during oddball experiments in which presentations of repetitive audio/visual stimuli sequences were intermittently interrupted by a deviant stimulus, implicating its involvement in signal detection and decision-making [57]. High levels of cortical spontaneous neuronal activity are observed in animals during natural sleep and this behavioral state is associated with global inhibition of the cerebral cortex to suppress consciousness, suggesting that neuronal activity observed during slow-wave sleep may be the basis for neuronal plasticity and to consolidate memory traces acquired during wakefulness [58]. The link between neural plasticity and slow waves is further supported by a recent human study in which intermittent θ burst stimulation inducing long-term potentiation in the left primary motor cortex in awake adults was followed by an increase of δ wave power in the same area [59].

The benefit of sleep in memory consolidation can be better appreciated from the perspective of slow-wave activity. Apparently, the number of neurons bursting in synchrony is directly correlated with the amplitude and slope of EEG slow waves. Moreover, this near-synchrony state is also directly related to the number of strength of synaptic connections among these neurons. Thus, per the synaptic homeostasis hypothesis, cellular homeostasis is restored and synaptic strength is renormalized via spontaneous slow-wave activity occurring during sleep [60]. Plasticity-dependent recovery could be improved by managing sleep quality, while monitoring EEG during sleep may help to explain how specific rehabilitative paradigms work [61].

γ power often increases during problem-solving, yet a 40 Hz frequency (γ band) is present during the rapid eye movement (REM) dream state sleep that interrupts the δ power-dominant slow-wave sleep [62,63], suggesting its role in modulating other oscillations. Given its omnipresence across different brain regions and its implication in a variety of cognitive function, the γ rhythm may serve to provide the synchronization between different neuronal networks [64,65]. High frequency oscillations, ripples in particular, play a crucial role in the information processing and consolidation of memory [66]. β power is observed in awake, attentive states that require working memory or it is found in the motor cortex during the preparation of movements [67]. It has been suggested that the function of the β oscillation could highlight a novel stimulus that would require further attention [68,69] based on its presence during novelty detection in the auditory system [70], reward evaluation [71], and sensory gating [68]. The hippocampal θ is also associated with memory function [72], as θ power increases during cognitive tasks as well as during verbal and spatial tasks due to an increase in memory load [73,74,75]. The α band is present in the occipital cortex during aroused states with eyes closed [63] or relaxed wakefulness. A form of α wave can also be observed during sensory, cognitive, and motor processes [34,57] and could play a role in the neuronal communication [76].

The reticular activating system (RAS), known as the arousal system, originates from the midbrain reticular formation and potentiates thalamic and cortical responses during both waking and REM sleep, a state of dream consciousness. Interestingly, clinical studies reported simultaneous changes between EEG and other vital physiological parameters including cardiorespiratory and blood pressure among the comatose patients [77,78], suggesting that there might be a common origin in the inherent periodicity of the arousal mechanisms. The RAS serves to modulate all the spectrum rhythms depending on sensory inputs and ongoing activity in the brain, in which ascending inhibition or decreasing excitation slow down the brain’s oscillations whereas excitation or disinhibition accelerates rhythms [79].

#### 3.2.2. In Animals

Ample experimental studies have focused on the understanding of oscillations in the hippocampus and corresponding behavior. For example, in the rat hippocampus, θ state occurs during walking, running, rearing, and exploratory sniffing, as well as during REM sleep [73,80,81,82]. Hippocampal θ is associated with stimuli in the working memory instead of the reference memory condition [73], thus it could be a tag for short-term memory [83]. Additional evidence also suggests that the hippocampal θ is associated with spontaneous movements in monkeys (7–9 Hz) [84] and locomotion in rodents [82]. Compared to hippocampal θ, the role of cortical θ is less clear. At least in cats, this rhythm is associated with task orientation during coordinated response, indicating its role in alertness, arousal, or readiness to process information [57]. The α frequency is present after sensory stimulation in the auditory and visual pathways, as well as in the hippocampus and reticular formation [57]. Although δ oscillation is dominant during the sleep state in animals [57], it is also observed during immobility and drowsiness in awake animals [80]. Sharp-wave associated ripples (SPW-Rs) are 100–200 Hz field oscillations with a duration of less than one second, present during awake immobility and slow-wave sleep in rat hippocampus and entorhinal cortex [66]. They are produced by inhibitory postsynaptic potentials (IPSP) occurring during bursts of interneurons, which converge on principal neurons and synchronize with the hippocampal sharp waves [85]. SPW-Rs play a critical role in memory consolidation and transferring memory from the hippocampus to the neocortex, of which the selective elimination during post-learning sleep resulted in the impairment of memory [86,87]. The γ wave has been commonly observed after sensory stimulation (auditory and visual) in the cortex, the hippocampus, the brain stem, and cerebellum in cats [57,88]. Interestingly, the γ amplitude in the rat hippocampus is larger during θ-associated behaviors such as exploration, sniffing, rearing, and the paradoxical phase of sleep than it is during non-θ-associated behaviors, suggesting that the γ oscillation is synchronized with the θ oscillation [89].

#### 3.2.3. Synchronized *vs.* Desynchronized Cortical State and Behavior

Apart from the conventional classification of brain activity based on frequency range, a new definition of the dynamics of network activity has emerged, known as the synchronized *vs.* desynchronized cortical states. A strong synchronization between the different networks consisting of both slow and large amplitude fluctuations as seen in slow-wave sleep is referred to as a synchronized state, characterized by up phases during which neurons fire, followed by down phases during which neurons are silent. The low frequency power is high (slow oscillation and δ oscillation), whereas the γ rhythm may decrease during this synchronized state. In contrast, the desynchronized state is present during waking or REM sleep, and it shows fast and low amplitude deflections during which the θ oscillations are dominant and the neurons fires continuously and irregularly without synchronization at the population level [80]. Between these two opposing brain states, there is a continuum of intermediate states with varying degrees of synchronization. The transitions between these two extreme states are mediated by neurotransmitters such as serotonin, noradrenaline, and acetylcholine that modulate the excitability of the neurons [90,91,92].

In general, the synchronized state is associated with immobility and quiescence in addition to slow-wave sleep and anesthetized state [93,94,95], albeit it is also present during waking. The amplitudes of oscillations in the synchronized state are usually smaller relative to those during slow-wave sleep [90,91]. Unlike the synchronized state, the desynchronized state is present in active and behaving rodents [96,97], and is often associated with an increase in the γ power among behaving animals [92], or during stimulation of subcortical structures [98] and attention [99]. However, some studies have shown contradicting results in which the γ power decreases in the desynchronized state [100,101].

Finally, it has been well documented that the EEG signal contains rich characteristics in its temporal, spectral, and spatial aspects that tightly correlate with behaviors. Behavioral state or brain state, as a loose term, is therefore often used to describe EEG patterns in various aspects that strongly correlate to a group of behavior (a.k.a. “state”) instead of to a limited set of performance (e.g., a sensorimotor task). For example, a strong oscillation at θ frequency (3–12 Hz) across the brain (particularly in the hippocampus and neocortex) has been referred to as a wakefulness state in both rodents [81,102] and humans [103], albeit with distinct electrophysiological characteristics between species such as central frequency, duration, and network coherence [103,104]. Accumulating evidence from human studies suggests that specific patterns (e.g., cross-frequency modulation, coherent network activity, *etc.*) during θ oscillation manifest cognitive processes [105,106,107]. Another example is a spectral change in the human motor cortex during motor movement [108], in which a decrease in power at a low frequency band (8–32 Hz) occurs with movement of a concomitant increase at a high frequency band (76–100 Hz). It is noteworthy that such spectral change occurs only at specific regions within the motor cortex, whereas the θ state (analogous to the desynchronized state) often involves multiple regions. In this regard, it remains unclear whether the movement-related spectral change is directly related to the θ state. Nonetheless, these region-, and behavior-specific changes of EEG may depict a general pattern when a certain kind of behavior (e.g., motor or cognition) is engaged.

## 4. EEG and the Cellular Origins of Oscillations

### 4.1. Under Physiological Conditions

#### Cellular Mechanisms

In order to delve further into the electrophysiological perturbations in response to stroke, we will first address the normal cellular mechanisms underlying the genesis of the electrical activity detected by EEG. The conventional EEG records the summation of currents of pyramidal neurons located at the surface of the scalp in the cortical layers. Similar to pacemaker cells, neurons are electrically excitable cells that can generate pulse and are able to propagate an incoming current via electrical and chemical signals sent from the axon of one presynaptic neuron to the dendrites of another postsynaptic neuron in a network. The neuron has a resting membrane potential of about −60 to −70 mV resulting from flux of ions in the neuronal environment. Neurons have high concentrations of potassium (K^+^) and chloride (Cl^−^) ions inside, while high concentrations of sodium (Na^+^) and calcium (Ca^2+^) ions are outside. These concentration gradients are maintained by a sodium-potassium pumping system. The closing or opening of ion channels induced by chemical or electrical stimuli modifies the flux of ions and leads to a modification of the membrane potential. An influx of positively charged ions into the cell reduces the charge separation across the membrane and results in a less negative membrane potential termed depolarization, whereas an efflux of positively charged ions increases the charge separation, leading to a more negative membrane potential called hyperpolarization.

Once activated, a neuron releases neurotransmitters into the synaptic cleft that either excite (depolarize) or inhibit (hyperpolarize) the adjacent postsynaptic neuron, depending on the nature of the neurotransmitters. Excitatory postsynaptic potential (EPSP) depolarizes the post-synaptic neurons resulting from the release of excitatory neurotransmitters such as glutamate or acetylcholine, while inhibitory postsynaptic potential (IPSP) hyperpolarizes neurons resulting from the release of inhibitory neurotransmitters such as γ-amino butyric acid (GABA) and glycine. An EPSP produces a flow of positive charges into the cell (current sink), while an IPSP acts in the opposite way by inducing a flow of positive charges out of the cell (current source). The summation of IPSP and EPSP induces a graded potential in the neuron so that when this membrane potential reaches the threshold potential, it induces an action potential that can propagate between neurons. The action potential is produced by a critical amount of Na^+^ entering in the cell and the opening of additional Na^+^ channels. This fast depolarizing event corresponds to the rising phase of the action potential, followed by the repolarization of the cell induced by an efflux of K^+^ ions and a decrease of Na^+^ influx. After an action potential, there is a refractory period during which another action potential cannot be generated due to a transitory inactivation of Na^+^ channels.

EEG detects field potential as IPSP or EPSP generated by neurons because those events are longer in duration than the action potential (up to 10 milliseconds *vs.* a few milliseconds). To summarize the mechanisms of current flow, EPSP that depolarizes the membrane results from excitatory currents, involving Na^+^ or Ca^2+^ ions, flowing inward toward an excitatory synapse (*i.e*., from the activated postsynaptic site to the other parts of the cell) and outward away from it. The outward current is referred to as a passive return current (from intracellular to extracellular space). IPSP, which hyperpolarizes the membrane, is caused by inhibitory loop currents that involve Cl^−^ ions flowing into the cell and K^+^ ions flowing out of the cell [20].

**Figure 1:**
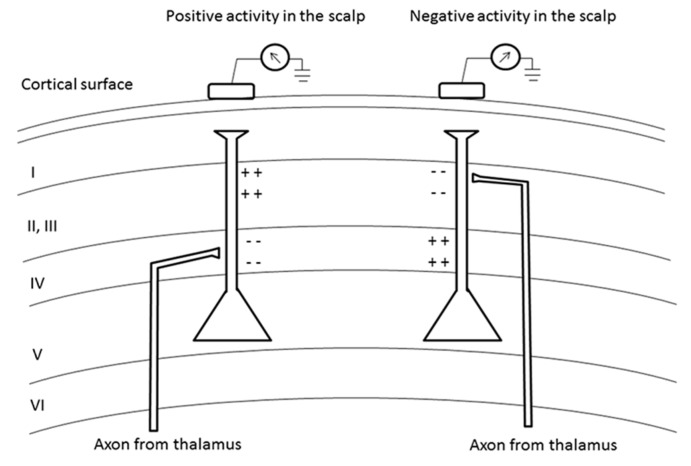
Generation of extracellular voltage fields. Relationship between the polarity of surface potentials and the location of dendritic postsynaptic potentials. EPSP depolarizing cell membrane induces a local negative local field potential (- -) and a positive local field potential (+ +) far away from the source. EPSP can also induce negative or positive activity in the scalp depending on the cortical layers excited.

The vertically orientated pyramidal neurons located in the cortex laminae are considered as a dipole that can generate extracellular voltage fields from graded synaptic activity. The dipole is created with a separation of charge vertically oriented in the cortex, and with apical dendrites extending upward to more superficial laminae and axons projecting to deeper laminae. The EEG detects the extracellular electrical fields generated closer to the cortical surface. The cortex is composed of several cortical laminae that can generate opposite current for the same synaptic event depending on the layer being excited. For example, an EPSP at the apical dendrite in layer II/III is associated with an extracellular negative field (active current field) and an extracellular positive field (passive current source) in the basal dendrite located in layer V. On the contrary, an EPSP on the proximal apical dendrite located in cortical layer IV is associated with an extracellular negative field (active current sink) and an extracellular positive field in the distal apical dendrite in layers II/III (passive current source) (Figure 1) [20]. Thus, a deep IPSP and a superficial EPSP will both generate a negative field in the scalp and *vice versa*. Therefore, a large population of neurons can be considered as a collection of oscillating dipoles [109].

The EEG tracings reflect the mean excitatory state of a pool of neurons rather than individual neurons, because the extracellular space beneath the electrode is traversed by currents from many cells. The interaction of signals of excitatory and inhibitory neurons explains why EEG waves oscillate [110], in which alternating rises and falls in amplitude come from negative feedback circuits formed by this complex interaction as the following: (1) the excitatory neurons are stimulated or cease to be inhibited; (2) the excitatory neurons stimulate the inhibitory neurons, dampening excitation; (3) the inhibitory neurons inhibit the excitatory neurons, reducing the electrical activity; (4) when the activity falls to a minimal level, the inhibitory neurons rest, releasing excitatory neurons from inhibition and the cycle resumes. In support of this conceptual framework depicting the collective activity underlying odor perception, another computational study further illustrates how synchronous rhythmic spiking in neuronal networks can be brought about by the interaction between excitatory and inhibitory cells in generating the pyramidal-interneuronal γ rhythm, in which the inhibitory neurons inhibit the pyramidal neurons that themselves project to the inhibitory neurons [111].

### 4.2. Under Pathological Conditions of Energy Failure

#### 4.2.1. Cellular Events after Ischemia

Because the pyramidal neurons located in the cortical layers III, V, and VI that generate graded EPSP and IPSP have been shown to be vulnerable to hypoxia and ischemia [112], we will discuss the cellular events occurring after ischemia and present evidence underlying the cause of EEG changes observed after stroke. Ischemia triggers an avalanche of cellular mechanisms that lead to short- and long-term consequences [113]. Given that neurons rely on adenosine triphosphate (ATP) as the main form of energy, a reduction of blood flow can significantly deprive brain cells of the glucose and oxygen necessary for the production of ATP. This reduction of oxygen activates the anaerobic glycolysis that produces lactate and the oxygen free-radicals burst, leading to ischemic damage and impaired electrical activity [114]. When the ionic gradients and the membrane potential cannot be maintained, it leads to the release of excitatory amino acids in the extracellular space and the accumulation of glutamate due to impaired reuptake by the transporters. The released glutamate activates the *N*-methyl-D-aspartate (NMDA) receptor that overloads the Ca^2+^ and causes an influx of Na^+^ and Cl^−^ into the neurons, leading to edema due to the passive diffusion of water into the cell.

As a universal second messenger, the overloaded Ca^2+^ activates proteolytic enzymes that degrade cytoskeletal proteins or extracellular matrix proteins. The generation of free radicals by the activation of the phospholipase via Ca^2+^ also produces membrane damage. Nitric oxide (NO) produced by Ca^2+^-dependent enzyme neuronal nitric oxide synthase (nNOS) forms peroxynitrite (reacted with a superoxide anion) that damages the tissue [115].

The ischemia-induced excitotoxicity has been well studied in the hippocampus and neocortex. In the CA1, short ischemia induces electrophysiological changes in pyramidal cells as a transient small depolarization followed by an increase in the excitability that leads to a hyperpolarization that changes the membrane resistance and abolishes the spontaneous or evoked spikes. Following ischemic reperfusion, the return of O_2_ and glucose induces a transient hyperpolarization before restoring to baseline conditions [113]. This post-stroke hyperexcitability is present during the first week to one month of recovery, and plays an essential role in post-stroke neuroplasticity. In rodents, it is manifested by expanded and less specific receptive fields as well as increased spontaneous activity [116,117]. This increased neuronal excitability has also occurred *in vitro* following oxygen-glucose deprivation, leading to the down-regulation of the GABAa receptor involved in the inhibitory pathway [118]. This hyperexcitability in surviving neurons contributes to a low frequency spontaneous activity (0.1–1 Hz) that fosters a permissive environment for axonal sprouting among rats with focal ischemia [119]. The modification of neuronal connections resulting from stroke-induced plasticity change in axons and dendrites [120,121,122] can persistently alter the generation and propagation of brain oscillations for weeks after stroke.

A variety of pathological states can cause aberrant changes in electrophysiology. For example, hypoxia induces a reversible hyperpolarization in the CA1 region of the hippocampus via a rise in K^+^ conductance. It has been shown that similar events are seen during hypoglycemia in the neocortex [123], the striatum [124], and substantia nigra [125], as well as in the hippocampus subregions such as CA1 [126] and CA3 [127] soon after the onset of ischemia [128,129]. Interestingly, hypoxia induces moderate depolarization instead of hyperpolarization [130] in some brain regions including the neocortex, dentate gyrus [131], striatum [124], and thalamus [112]. It has been shown that inducing anoxia with cyanide can depolarize or hyperpolarize the same CA1 neuron depending on its resting potential [132], providing the neural basis for the diverse EEG changes seen after stroke.

#### 4.2.2. Cerebral Blood Flow (CBF) and EEG

Due to the great complexity and variation in brain ischemia-induced pathophysiology, a general consensus regarding the modifications of the brain oscillations after stroke is hard to reach, except that the type of electrical activity appears to correlate with cerebral blood flow [133,134,135,136], oxygen, and glucose levels [137,138].

EEG abnormality begins to emerge when the CBF decreases to 25–30 mL/100 g/min compared to the normal range of 50–70 mL/100 g/min. [134]. Table 1 illustrates the critical levels of CBF for categorical reduction or loss in EEG amplitude and frequency, with corresponding changes in cellular metabolism and neuronal morphology [133,135,138,139]. When CBF falls below 18 mL/100 g/min, it crosses the ischemic threshold and induces neuronal death. When it reaches 12 mL/100 g/min or below, infarction becomes evident because of the progressive loss of transmembrane potential gradients of neurons. If the CBF is below the ischemic threshold but maintained above the infarction threshold, the effect on metabolism or cell survival is still reversible, with visible electrical activity as δ oscillations. When the CBF falls below the threshold of infarction for a substantial amount of time, specifically for more than 45 min at 14 mL/100 g/min or less, the spontaneous neuronal activity never returns, even after reperfusion, and the damages is irreversible [114,133,140,141].

While CBF is directly correlated with brain oscillations, it has been shown that the glutamate concentration (excitatory neurotransmitter) is associated with the θ waves (4–7 Hz) in the frontal lobe and the hippocampus during cognitive tasks in humans [142]. Abnormal release of glutamate coincides with CBF levels of 20–30 mL/100 g/min and is associated with peri-infarct depolarization [140,143]. Parallel experimental data show that a reduction in EEG power across all frequency ranges 1–3 h after permanent middle cerebral artery occlusion (pMCAO) in the ischemic ipsilateral cortex of rats is associated with a decrease of 30% of CBF compared to baseline and an increase of 1400% of glutamate release [144]. Moreover, CBF and the cerebral rate of oxygen metabolism studied with Xenon computed tomography and positron emission tomography show that regional EEG changes reflect the coupling of CBF and metabolism in ischemic stroke [145]. In early subacute stroke, the EEG correlates with the CBF because the oxygen extraction fraction increases to preserve the cerebral rate of oxygen metabolism (also known as misery perfusion or stage 2 hemodynamic failure). During the period of luxury perfusion or stage 3 hemodynamic failure, the EEG is no longer correlated with the CBF but instead with the rate of cerebral oxygen metabolism [145,146]. It should be noted that the cellular damages such as decreased protein metabolism and neuronal death appear even before the critical stage of CBF in the peri-infarct area [140].To recapitulate, increased power in slower frequency bands (as θ or δ) and decreased power in faster frequency bands (as α and β) are seen with the reduced rate of cerebral oxygen metabolism [145]. Second, the δ rhythm seems to be the most reliable parameter correlating with CBF and metabolism changes during focal ischemia.

**Table 1:** Physiological coupling among cerebral metabolism, EEG, and cellular response, and the consequence on neuronal injury. EEG: electroencephalography, CBF: cerebral blood flow, ATP: adenosine triphosphate.

CBF Level (mL/100 g/min)	EEG Abnormality	Cellular Response	Degree of Neuronal Injury
35–70	Normal	Decreased protein synthesis	No injury
25–35	Loss of fast β frequencies and decreased amplitude of somatosensory evoked potentials	Anaerobic metabolism Neurotransmitter release (glutamate)	Reversible
18–25	Slowing of θ rhythm and loss of fast frequencies	Lactic acidosis Declining ATP	Reversible
12–18	Slowing of δ rhythm, increases in slow frequencies and loss of post synaptic evoked responses	Sodium-potassium pump failure Increased intracellular water content	Reversible
<8–10	Suppression of all frequencies, loss of presynaptic evoked responses	Calcium accumulation Anoxic depolarization	Neuronal death

#### 4.2.3. Penumbra and Core

The ischemic territory is not homogenous in many aspects due to the variation of the hemodynamics. The core is supplied with a 20%-below-normal level of cerebral blood flow and neuronal survival is threatened by acidosis, lipolysis, proteolysis, and disaggregation of membrane microtubules after the bioenergetics failure and the ion homeostasis breakdown. Besides, because of the K^+^ and glutamate release, the neurons depolarize but cannot repolarize. Unlike the core, neurons in the penumbra struggle to maintain function but exhibit perturbed electrical activity due to partial energy metabolism preservation. Since repolarization of neurons following depolarization consumes energy, the succession of “peri-infarct depolarization” occurs at the expense of the valuable and scarce energy remaining in the penumbra, leading to a perpetual depletion of the energy, and hence, a further expansion of the core and penumbra [115]. To further illustrate the vulnerable and dynamic state of the peri-infarct penumbra, a recent study elegantly demonstrated that supply-demand mismatch transients triggered peri-infarct depolarizations (PIDs), a phenomenon akin to spreading depression (SD) frequently occurring in experimental and human stroke [147,148]. SD can be detected by changes in electrical activity, ionic potential, or optical signal, and is specifically seen as propagating waves of suppressed electrocorticogram (ECoG) activity, direct coupled (DC) potential shift by two serial intracortical microelectrodes sensitive to ionic changes, or spreading pallor in time-lapsed images during intrinsic optical imaging [147,148]. In principle, factors causing regional pO_2_ to drop below the depolarization threshold within a penumbra hot zone can trigger PIDs, including hypoxia or hypotension. For example, sensory stimulation of the susceptible hot zone by tactile stimulation of the forelimb increased O_2_ extraction and supply-demand mismatch, increasing the metabolic burden, triggering anoxic depolarization, and worsening tissue perfusion and ischemic outcome. Interestingly, the somatosensory stimulation-induced PIDs were prevented by normobaric hyperoxia. Induced hypotension via controlled blood withdrawal also triggered PIDs, which did not require cortical neuronal activation, nor could they be inhibited by tetrodotoxin (TTX) [147,148].

The nature of the perturbation in brain oscillation can provide insight into the pathophysiology and evolution of the ischemic core and penumbra. For example, patients with acute unilateral ischemic stroke in the MCA territory experience an increase in δ activity (low frequency band), whereas there is a decrease in α activity (high frequency band) in the ipsilateral parieto-occipital cortex and the contralateral medial and posterior cortex [149], reflecting the state of brain metabolism as well as neural activity in the core and penumbra, respectively [150,151]. Consistent with this concept, the power of high frequency oscillation like the β band was found to decrease proportionally with the size and proximity of the infarct in patients one day after stroke [152]. However, as an exception to the rule, penumbra could also generate slow activity like δ or θ [153].

Alternative interpretations regarding the origin of the slow frequency activity after brain ischemia have emerged since the witness of a δ variant known as the polymorphic δ activity. The core support for the alternative theory derives from the fact that a direct lesion to the cortical gray matter alone did not produce slow-wave activity due to the coincidental destruction of the neuronal generators located in the cortex; hence, a lesion in the subcortical white matter induced irregular δ activity in the cortex overlying the infarct [154]. Evidence suggests that the polymorphic δ activity is cortical and it results from a disruption of corticocortical and thalamocortical connections [155], since the deafferentation of cortical neurons with thalamal lesions led to the increase of δ-like activity in the unilateral or bilateral cortex, bilateral hypothalamus, or bilateral mesencephalon [154,156]. Furthermore, surface positive δ waves may represent an inhibitory phenomenon such as a hyperpolarization, based on the following possibilities: (1) the presence of synaptic IPSPs at the soma or basal dendrites; and (2) an influx of the calcium mediated by the efflux of potassium after hyperpolarization. Given the fact that the administration of cholinergic antagonist atropine led to polymorphic δ activity, the apparition of the slow-wave activity or the increase of the power of δ after stroke could result from an impairment of the cholinergic pathways [157].

To summarize, the EEG changes observed after ischemia are caused by an electrical impairment of the neurons due to the changes of the membrane potential induced by energy deprivation. This energy deprivation results from the reduction of the CBF and leads to irreversible neuronal damages if the CBF is not restored in time. However, the neuronal origin of the increase of slow or δ oscillations and the decrease of high frequency oscillations after stroke is still under debate.

## 5. EEG in Stroke Conditions

Evidence suggests that ischemic stroke, a direct consequence of CBF impairment in local cerebral areas, is associated with brain oscillation fluctuations. Due to the non-invasive and real-time nature of the technique in recording the changes in brain activity, EEG has been widely employed in both the clinical and research fields. A wealth of information regarding the modifications of the brain activity observed after stroke has been catalogued and potential electrophysiological biomarkers diagnosing stroke, monitoring treatment response as well as secondary adverse events, or predicting the post-stroke outcome have emerged.

### 5.1. Modifications of the Brain Oscillations in Experimental Stroke

A recent comprehensive review documented the EEG changes commonly observed after focal cerebral ischemia in rodents [158]. In essence, during the acute phase of ischemia in a transient MCAO model, the distribution of the power of the EEG spectrum (0–30 Hz) after Fourier transformation in animals is as following: 85% of δ oscillations, 7% of θ oscillations, 5% of α oscillations, and 3% of β oscillations. Thus, ischemia has resulted in an increase of low frequency and a decrease of high frequency oscillations, or specifically a decrease of the α-to-δ ratio [17,159], considering the baseline distribution as 25%–45% of δ, 40% of θ, 12%–15% of α, and 3%–20% of β oscillations [16,17]. In particular, an increase in δ power in the ipsilateral hemisphere after transient MCA stroke was reported in both the subacute and chronic phase from 24 h to seven days or beyond [16,17,160,161,162,163]. Another study reported that an increase of the ipsilateral δ and θ power occurred as early as one minute following intraluminal filament occlusion of the proximal part of MCA that leads to impairment in the subcortical brain regions [164]. The increase of both δ and θ activity was also reported eight days after tMCAO in rats in the fronto-parietal, occipital, and temporal regions, whereas α and β activity were depressed [165]. Diaschisis frequently occurs after focal brain ischemia [166,167], of which the transhemispheric diaschisis refers to changes in the contralateral hemisphere detected after unilateral stroke [168]. Some studies suggest that an increase of the δ activity in the contralateral sensorimotor cortical areas correlated with an ipsilateral increase one to seven days after MCAO in rodents [16,159,161,169]. On the other hand, other studies have shown that an increase in the contralateral EEG power in the somatosensory cortex accompanied a suppression of the EEG activity in the ipsilateral side 15 min after tMCAO in rats. Due to the lack of consensus in the evolution of the contralateral side, an asymmetric index is often used to reflect changes of rhythms in both hemispheres over time. This asymmetry calculated by the brain symmetry index (BSI) or the global pairwise derived brain symmetry index (pdBSI) is also present in experimental studies as reported during both acute (1 h post-stroke) and chronic phases (up to 14 days post-stroke) in young and one-year-old rats, respectively [161].

The literature is less clear concerning the modifications of the power of γ, β, and α bands. In general, these three bands decrease after stroke in rodents, although contradicting results do exist. For example, a 35% reduction of the amplitude of α waves and β waves in the ipsilateral hemisphere was reported three to seven days after tMCAO [158,160]. The α band power decreased from day one to day 28 after pMCAO [158,170], whereas other studies reported an increase of δ, β, and rhythmic α activity by seven days in the contralateral cortex after stroke in a rat model of tMCAO [16]. Since γ oscillations have been implicated in higher cognitive function and might depend on the mitochondrial redox state, they are highly sensitive to decreases in pO_2_, and are thus likely to be susceptible to the reduction in blood flow [171,172].

Some evidence seems to implicate that an increase of the infarct volume is correlated with an increase of the δ power and neurological deficits [159,173]. The volume of infarction is also correlated with the acute δ change index [174], pdBSI [175], relative α percentage, relative α-β percentage, relative δ-θ percentage, δ/α ratio, or δ-θ/α-β ratio [150]. It is likely that the loss of the fast frequencies and the increase of slow-wave activity are caused by the pathological neural tissue, leading to an impairment of the communication in the affected network regions [154].

### 5.2. Clinical Applications of Continuous EEG Monitoring during Acute Ischemic Stroke

In contrast to computed tomography (CT) or magnetic resonance imaging (MRI), EEG is inexpensive, less invasive, widely available, and above all, it can detect changes of brain electrical activity within minutes of stroke onset even in the conditions of sleep, sedation, or loss of consciousness [133,176]. To attest to the sensitivity of EEG, previous studies showed the efficacy of emergency EEG to detect ischemic changes in patients with no abnormality in the initial CT scan [177,178]. Recent advances in computer technology enable us to monitor EEG anytime and anywhere by using downsized and manageable portable EEG devices. This would be helpful for non-neurologists at the point-of-care, especially in conditions like transportation of patients by ambulance, initial assessment by paramedics, or making diagnoses in hospital facilities with no availability of CT or MRI.

Complementary to experimental findings, extensive studies in humans have been conducted to correlate EEG changes with the size of the lesion or the location of the ischemic infarct [179]. Unlike the aberrant changes commonly seen in large acute strokes, EEG often is normal or shows subtle focal θ activity in lacunar infarcts [180], further supporting the coupling between CBF and EEG patterns. Sometimes focal slow-wave activity as the δ rhythm in awake adults, which could result from deafferentation of subcortical structures, indicates a localized structural lesion [181]. Nonetheless, continuous slow-wave activity is more representative of severe brain damage, whereas intermittent slow activity is representative of smaller lesions [156]. In addition to subcortical infarct such as the lacunar stroke, EEG may also show reduced sensitivity in patients with posterior cerebral artery (PCA) infarct [177,182,183]. Although some recent studies suggest that EEG is useful in all types of ischemic stroke regardless of ischemic location [184], it seems still difficult to detect a transient ischemic attack (TIA) by EEG [185]. EEG also can also predict some adverse events like delayed cortical infarct in subarachnoid hemorrhage (SAH) [186,187], or severe edema in malignant MCA infarction [188,189]. Table 2 summarizes some major characteristics associated with subtypes of ischemic stroke including location and clinical conditions from selected literature.

**Table 2:** EEG characteristics in various locations and subtypes of ischemic stroke.

Stroke Subtypes	Summary	Time Frame of EEG Detection Relative to Stroke Onset	EEG/qEEG Characteristics
Large (Cortical, including ACA, MCA, PCA territories)	EEG abnormalities following cortical infarction depended on infarct location	<2 weeks (<24 h (34%), <1 week (50%))	Lateralized EEG abnormalities 80% in MCA territory, 86% in cortical watershed zone, but 50% in PCA territory [177]
	Strong association between EEG mapping of δ power and lesion locations by CT	<24 h	Close correlation between EEG abnormalities (increased δ power) except striatocapsular in 85% patients [182]
	EEG monitoring is useful in all ischemic strokes regardless of locations. Also, pdBSI predicted radiologically (CT, MRI) confirmed stroke with an accuracy higher than the National Institute of Health stroke score (NIHSS) score at admission	<7 days (<72 h (81%))	Increased pdBSI, DTABR, even in PCS and LACS [184]
Small (subcortical, lacunar)	EEG has relatively low sensitivity in patients with subcortical infarcts	<2 weeks (<24 h (34%), <1 week (50%))	82% normal or non-lateralized EEG changes in subcortical lesions [177]
	EEG has relatively low sensitivity in patients with first lacunar infarcts	<7 days	Abnormal EEG in 43% patients with first lacunar stroke [183]
	EEG abnormalities depend on affected lesions in subcortical regions	<24 h	Normal EEG in striatocapsular regions 70% abnormal EEG in other subcortical regions [182]
TIA	EEG has low sensitivity in patients with TIA	<24 h	Non-significant difference between TIA and control by using pdBSI and DTABR [185]
DCI in SAH	ADRs may allow earlier detection of DCI in patients with severe SAH	Post-operative day two to post-SAH day 14	ADR decrease in patients with DCI [186]
	EEG changes preceded detection of vasospasm/DCI in standard procedures by 2.3 days	2–12 days (median 5.2 days)	Decrease in α or θ power few days before vasospasm/DCI [187]
Malignant MCA infarction	Emergence of high-voltage contralateral hemisphere δ activity might represent midline shift due to substantial edema in ipsilateral hemisphere and increased intracranial pressure	<25 h	Increasing δ power in contralateral hemisphere in malignant course [188]
	EEG and brain stem auditory evoked potentials have prognostic value for patients who develop malignant edema	<24 h	Diffuse generalized slowing and slow δ activity in the ischemic hemisphere pointed to a malignant course [190]

Abbreviations: CT: computed tomography; MRI: magnetic resonance imaging; qEEG: quantitative electroencephalography; ACA: anterior cerebral artery; MCA: middle cerebral artery; PCA: posterior cerebral artery; ACS: anterior circulation syndrome; POCS: posterior circulation syndrome; LACS: lacunar syndrome; DCI: delayed cerebral ischemia; SAH: subarachnoid hemorrhage; ADR: α/δ ratio; DTABR: (δ + θ)/(α + β) power ratio; pdBSI: pairwise derived brain symmetry index; TIA: transient ischemic attack; CT: computed tomography; MRI: magnetic resonance imaging.

Apart from the generalized or regional bisynchronous slow activity or generalized asynchronous slow activity, other EEG changes after stroke include focal attenuation of a specific rhythm, usually the faster activity frequencies, as well as general attenuation or suppression of one or multiple brain oscillations [179]. Besides the fact that both the repartition of the band and the power between each wave changes, there is an apparition of abnormal patterns in stroke patients [179] and in animal models of MCA stroke [158]. The abnormal patterns can be attributed to non-convulsive seizures, occasional rhythmic spike-and-wave or polyspike discharges, polymorphic slow-wave δ activity, intermittent rhythmic δ activity associated with a 4–7 Hz range large-amplitude burst, periodic lateralized epileptic discharge, rhythmic discharges with a 1–4 Hz frequency spike, recurrent sharp or slow waves every 1–8 s, and pathological high frequency oscillations.

### 5.3. Continuous EEG Monitoring during Thrombolysis

One report using continuous EEG showed a prompt reduction of δ power before symptomatic recovery within 20 min after intravenous tissue plasminogen activator (IV tPA) administration and persisted for at least three months [191]. Another study of 16 patients with tPA treatment showed a significant correlation between changes in BSI and neurologic recovery by using National Institute of Health stroke score (NIHSS) [192]. Moreover, one case report showed that two days after treatment with tPA, there was a resolution of pre-tPA δ activity correlated with an improvement of neurological deficits and complete recanalization of occluded MCA by using MR angiography [193], though this study did not report changes of EEG soon after tPA administration. These studies may indicate indirectly that continuous EEG monitoring could provide real-time information about successful recanalization by IV tPA and this could be important information for making a decision about additional treatments such as intra-arterial thrombolysis or mechanical thrombectomy. A future EEG monitoring study combined with intra-arterial therapy may clarify more detailed EEG changes before and after recanalization and enhance the utility of continuous EEG monitoring during IV tPA therapy. Continuous EEG monitoring also may detect not only improvement but also serious secondary adverse events, such as massive hemorrhagic transformation, severe cerebral edema, restenosis or reocclusion after recanalization therapies, l in real time. Apart from the potential in early detection of secondary events, other reports indicate that continuous EEG may also provide information for early diagnosis of other stroke conditions like a TIA [153] or delayed cerebral ischemia in SAH patients [186,187].

### 5.4. Biomarkers of Prediction after Stroke

Real-time EEG during and after acute stroke has become not only an invaluable tool to diagnose, but also to predict the evolution and outcome of stroke as an electrophysiological biomarker. Global changes such as loss of reactivity [194] or absence of sleep-wake cycle [195] constitute a bad prognosis and may implicate the presence of brainstem impairment due to its close relationship with the cortical layers. A unilateral prominent slow δ or a decrease of α is also a sign for poor outcomes [196]. In contrast, good outcomes are correlated with the lack of δ and presence of faster frequencies within 24 h in regional changes [189].

The severity of stroke as assessed by the NIHSS in acute and subacute periods in humans is found to correlate with some derived EEG parameters such as the brain symmetry index (BSI) [192,197], the global pairwise derived brain symmetry index (pdBSI), the relative α percentage, the relative δ-θ percentage, the relative α-β percentage, the δ-α ratio, and the δ-θ/α-β ratio [151,175,198]. A positive correlation was found between an increase of δ power during acute stroke and in patients with severe stroke including those with worse NIHSS scores eight months after stroke [153,189,199]. High asymmetry in the BSI during the acute phase is also associated with poor outcomes [153,197], as in the case that a post-stroke shift of scalp δ power maxima from the ipsilateral hemisphere to the contralateral hemisphere indicated substantial worsening of cerebral pathophysiology. For example, high δ power was detected during the eight-hour post-stroke period in the fronto-central and fronto-temporal electrodes in the ipsilateral side, followed by high δ in contralateral side 16 h post-stroke. This high δ remained 25 h post-stroke whereas the δ power decreased in the ipsilateral side. It is noteworthy that the patients who had an important δ shift died in the ensuing days [188], suggesting the prognostic value of δ EEG changes.

Another study reported that poor recovery was associated with increased power in δ and θ bilaterally four to ten days after unilateral acute stroke in the MCA territory, in conjunction with increased power in β and γ in the contralateral hemisphere [200]. Patients with unilateral ischemic stroke in the middle and/or anterior cerebral artery show the α band locally reduced in brain regions critical to observed behavioral deficits three months after stroke [201]. Moreover, a high δ/α power ratio [198] measured during subacute stroke is associated with high scores of NIHSS at 30 days post-stroke, indicating bad outcomes. Conversely, an absence of slow activity with minimal decrease in other background frequencies predicts good outcomes (95% of success), whereas bad outcomes are predicted by continuous polymorphic δ and a decrease of the α and β activity in the ischemic hemisphere (79% of success) [196].

Although the occurrence of slow waves after stroke was often associated with adverse consequences of stroke and even used as a predictive biomarker of post-stroke outcomes, this group of oscillations has also been considered as a marker of neuronal plasticity. Among these, axonal sprouting has been regarded as an important component of functional plasticity and recovery following central nervous system (CNS) injury including stroke [202]. Following thermal ischemic lesion in the somatosensory cortex, synchronous neuronal activities were found in the perilesion cortex with a frequency range of 0.2–2 and 0.1–0.4 Hz on day one and days two to three after ischemic injury, respectively. Inactivating the latter slow-wave pattern in the perilesion area by using TTX blocked axonal sprouting, suggesting that the δ oscillations observed in the perilesion cortex can be a lesion-induced signal for anatomical reorganization within the brain [119]. The link between slow-wave activity and post-stroke neuroplasticity is further supported from the perspective of slow-wave sleep [61]. Mice treated with γ-hydroxybutyrate, a drug used to promote slow-wave sleep in humans, showed a faster recovery in motor function after stroke [203]. In addition, sleep disruption not only negatively impacted post-stroke functional recovery, but also specifically impaired processes associated with functional recovery including axonal sprouting and neurogenesis [204,205].

To conclude, when the rate of cerebral oxygen metabolism is reduced, there is an associated increase in the δ and θ frequency oscillations (lower frequencies) and a decrease in faster frequencies such as β and α [145], although the δ wave change appears to be the more reliable index for the reduction of CBF and brain metabolism during focal ischemia. Moreover, using global parameters such as the α-β/θ-δ power ratio in order to detect and predict early and subtle ischemic EEG changes seems to be appropriate [145,206,207].

## 6. EEG, Oscillations Coupling and Perspectives

Despite the variation in findings, findings in global EEG changes after stroke coalesce to an increase of slower frequency oscillations and a decrease of faster ones. However, the relationship between the contralateral hemisphere and the ipsilateral hemisphere with respect to electrical activities and their temporal evolution remains controversial. Similarly, at the cellular level, the decision for neurons to depolarize or hyperpolarize hinges on the state of resting potential even under the same condition. Apart from the biological variation to ischemia, a great deal of the variability in results can be attributed to the complex connections that propagate electrical signals, and the cerebral cortex is the very source of signals recorded in human EEG. The complexity of ipsilateral cortical connectivity is best exemplified by the barrel field somatosensory cortex that receives projections from the motor cortex, frontal cortex, and other parts of the somatosensory and parietal cortex via layers I and II/III. [208]. Cortical neurons also project to the contralateral hemisphere via the callosal neurons in layers II/III, IV, and VI. The synchronization between the homotopic areas in two hemispheres is interrupted after lesion of the corpus callosum [209]. For the subcortical inferences, we can cite the thalamus, the hypothalamus, and the basal nucleus among all the other subcortical structures projecting to the neocortex.

In light of the continuum represented by brain oscillations, using the conventional approach by treating them as individual “explicit” entities seems to reach an impasse for advancement. The shifting between oscillations under conditions of low blood flow and the detection of polymorphic δ variant are particularly insightful in this regard. Furthermore, the ability of one oscillation in modulating another across brain regions adds even more dimensions to the already complex relationship. Since low frequency waves propagate more than high frequency ones that tend to stay localized to small structures [18,19], θ and δ waves are found to propagate through the entire brain as directional waves, whereas α, β, and γ waves are localized and driven by θ and δ. Ample studies sought to understand the interaction between γ and θ oscillations. For example, it has been shown that neocortical neurons were modulated by the hippocampal θ rhythm, with increased firing when the phase of θ is down in the CA1. Interestingly, a greater proportion of interneurons, e.g., 32% in the parietal cortex and 46% in the prefrontal cortex were modulated by θ waves compared to that in pyramidal neurons (11% in the parietal cortex and 28% in the prefrontal cortex) [24]. Another study demonstrated that the γ oscillation was phasically modulated by the θ cycle and the amplitude of γ oscillation varied as a function of the θ cycle. Moreover, the amplitude of γ activity was larger and the hippocampal interneurons in the hilus of the dentate gyrus fired rhythmically with a higher rate during θ-associated behaviors such as exploration, sniffing, rearing, and the paradoxical phase of sleep. It should be mentioned that after entorhinal cortical lesion, the amplitude of the hippocampal θ (5–10 Hz) decreased by 50%–70% and the frequency of γ oscillations reduced in the dentate gyrus from 40–100 to 40–60 Hz [89]. Additional studies further suggest that the γ oscillation in the cortex is driven by θ oscillation from the hippocampus [24,89,210].

**Figure 2:**
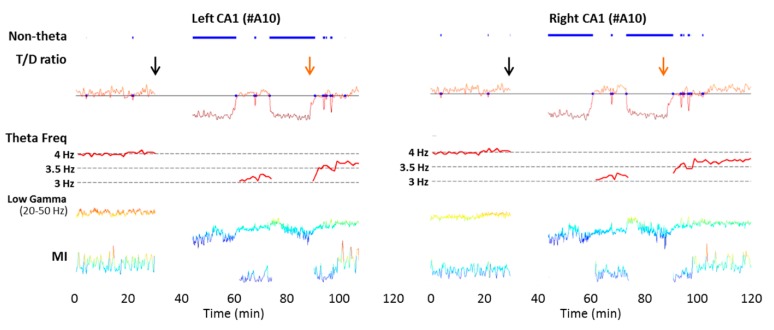
Acute cortical ischemia induces a reduction in the hippocampal θ frequency and the θ/δ ratio. Extracellular recordings were performed using multisite silicon probes (A1X16-5mm-100-703, NeuroNexus Technologies) under urethane anesthesia for 2 h. Data from the channel located at the stratum lacunosum moleculare were used for the analysis based on the high signal-to-noise ratio of θ and low-γ oscillations at the molecular layer compared to other hippocampal layers. Experimental stroke was induced by a permanent occlusion of the left, distal MCA and temporary occlusion of the bilateral common carotid arteries (CCAs) for 60 min. An immediate transition to slow-wave sleep from θ state occurred after MCAO, followed by the return of the θ state after reperfusion. Reductions in θ frequency, θ/δ (T/D) ratio, and modulation index between θ and low γ (MI_Low γ_) and a decrease in low γ power were evident during some periods of occlusion and reperfusion. MI was computed based on Tort *et al*., (2010) with the band-pass filter set at 20–50 Hz [211], corresponding to the low-γ power modulated by the θ phase. Color: relative values of low-γ power or modulation index (warmer color reflects larger value). Black arrows: stroke onset at 30 min; orange arrows: start of the reperfusion of the bilateral common carotid arteries at 60 min after stroke. Blue line: Non-theta periods. Note: recording of the initial period after MCAO was temporarily interrupted due to ischemic surgery.

Evidence showing modulation between other oscillatory bands has just begun to emerge. A recent study investigated how slow activities such as δ rhythm coordinate fast oscillations such as γ rhythm over time and space. The study recorded the local field potentials in the cortico-basal ganglia structure of freely moving, healthy rats and showed that the phase of δ waves modulates the amplitude of γ activity [212]. The complexity of the relationship between various band frequencies and how it can be modified under pathological conditions is best exemplified in the α wave in the thalamus. An increased depolarization in the thalamocortical neurons that discharge in the range of 2–13 Hz can lead to oscillation in the α frequency (8–13 Hz), while a reduced depolarization of the same neuronal subpopulation gravitates brain waves towards the θ rhythm (2–7 Hz) [213]. Modification in oscillation coupling has indeed been reported in pathological conditions including schizophrenia, Parkinson’s disease, or autism [214]. Given that θ-γ coupling seems necessary for working memory [215] and that working memory is disturbed in stroke patients [216], it is surprising that there is no evidence showing an impaired θ-γ or other oscillatory couplings in human or experimental stroke. Some factors might have contributed to the paucity of data in this area; for example, θ phase calculation relies on the sinusoidal assumption, while human θ (either EEG or hippocampal θ) is not sinusoidal-like. Although rodent θ is sinusoidal and an increase in δ power does occur after experimental stroke, deciphering clear θ epochs from other frequency bands is no easy task. In addition to technical constraints, recording human hippocampal θ is rare and not favored in the clinic due to its risk. Nonetheless, using a rat model of MCA stroke with injury restricted to the parietal cortex, we found that stroke caused (1) an immediate transition to the slow-wave sleep state; (2) a decrease in low-γ power; and (3) a decrease in θ frequency in the hippocampus, a brain region remote from the ischemic site that shows no structural damage (Figure 2). It also appeared that in the ipsilateral hippocampus, the modulation index (as a measure of the strength of the θ phase modulating the low-γ power) was reduced in the initial first hour after stroke onset. Following reperfusion of the common carotid arteries (CCAs), low-γ power remained to be reduced, suggesting a disrupted connectivity between the cortex and the hippocampus necessary for processing spatial information.

## 7. Conclusions

In summary, although the quest to understand the electrical activity in the brain commenced more than a century ago, ever-growing endeavors in this area continue to thrive upon the improvement of technology. In light of the continuum in brain oscillations in the spectrum domain, it seems futile to attribute the behavioral states, anatomical structures, or even cellular mechanisms exclusively to a single, specific frequency band. Nonetheless, with some exceptions, a general consensus is reached that an increase in the slow band frequencies, referred to as slow oscillation and δ oscillation, is associated with not only the slow-wave sleep state but also brain ischemia. Conversely, high band frequencies, such as the α, β, and γ oscillations, are associated with awake states or cognitive task engagement, and their presence frequently reduces after stroke. To harmonize with the various physiological states such as the wakefulness phase and sleeping phase, the mammalian brain rhythms are modulated according to the degree of arousal. The oscillations in the membrane potential may underlie the coherent responses of cortical and thalamic neurons to communications from the outside world during awake states and from inside during sleep. Since all the cortical rhythms are modulated by the ascending brainstem reticular-activated system, it nominates the thalamus as a potential candidate for the supervision of the electrical activity in the brain. The immediate EEG changes observed after stroke are a direct consequence caused by the reduction of the cerebral blood flow that later results in neuronal impairment or neuronal death. This cellular impairment in turn leads to a disorganization of the electrical activity that is reflected by the global EEG changes. Individual or derived EEG parameters have been insightful in the diagnosis of ischemic stroke and prognosis of the outcomes after stroke. The utility of EEG as a potential biomarker for stroke outcome and therapeutic efficacy warrants more validation. 
